# Sulforaphane as a promising anti-caries agents: inhibitory effects on *Streptococcus mutans* and caries control in a rat model

**DOI:** 10.3389/fmicb.2024.1427803

**Published:** 2025-01-03

**Authors:** Meijiao Yu, Yu Chen, Sishi Dong, Zhongxin Chen, Xuelian Jiang, Yufei Wang, Linglin Zhang

**Affiliations:** ^1^State Key Laboratory of Oral Diseases & National Center for Stomatology & National Clinical Center for Oral Diseases, West China Hospital of Stomatology, Sichuan University, Chengdu, Sichuan, China; ^2^Department of Cariology and Endodontics, West China Hospital of Stomatology, Sichuan University, Chengdu, Sichuan, China

**Keywords:** dental caries, sulforaphane, isothiocyanates, *Streptococcus mutans*, bacteriostatic therapy, natural products

## Abstract

Dental caries has been one of the most prevalent diseases globally over the last few decades, threatening human oral and general health. The most critical aspect in caries control is to inhibit the dominant cariogenic bacteria *Streptococcus mutans* (*S. mutans*). Sulforaphane (SFN), a compound found in a wide range of cruciferous plants, has demonstrated bacteriostatic activities against various pathogenic bacteria. The objective of the present study was to investigate the effects of SFN on *S. mutans* though both *in vitro* and *in vivo* experiment. The minimum inhibitory concentration (MIC) against *S. mutans* was determined at 256 μg/mL. The growth of *S. mutans* and the biofilm formation were inhibited by SFN in a dose-dependent manner through suppressing the synthesis of extracellular polysaccharide (EPS) and acid production, as well as decreasing the acid tolerance. Meanwhile, SFN significantly weakened the cariogenic properties of *S. mutans* at sub-inhibitory concentrations, which were further illustrated by quantitative real-time PCR (qRT-PCR). Moreover, SFN were found to inhibit quorum sensing (QS) by downregulate *comCDE* system in *S. mutans*. Further investigation using a rat caries model displayed a prominent caries control in the SFN-treated group with no observed toxicity. The notable results demonstrated in this study highlight the potential of SFN as a natural substitute for current anti-caries agents, while also providing valuable insights into the potential applications of SFN in caries control.

## Graphical abstract

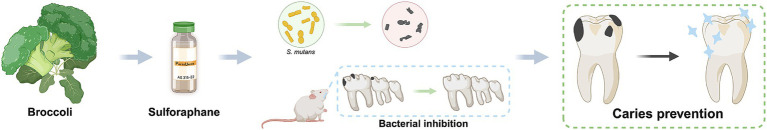

## 1 Introduction

As one of the most prevalent oral diseases in the world, caries has been extensively studied in the past few decades. It is acknowledged as a chronic and progressive destruction of the dental hard tissues that occurs under the influence of a variety of factors dominated by cariogenic bacteria (Machiulskiene et al., 2020). Among various pathogenic bacteria, *Streptococcus mutans* (*S. mutans*) plays the most crucial role in the occurrence and development of caries (Lemos et al., 2019). *S. mutans* exhibits a strong ability to adhere to tooth surfaces and tendency to produce intracellular polysaccharides (IPS) and extracellular polysaccharides (EPS), which facilitate the bacteria to metabolize carbohydrates and generate acid. Moreover, the robust acid resistance of *S. mutans* allows for the development and maintenance of the cariogenic state in dental plaque biofilm within an acidic environment (Zheng et al., 2023; da et al., 2021). Due to the protective effect of the plaque biofilm matrix, the acid produced is concentrated on the surface of tooth, resulting in localized pH reduction and subsequent tooth demineralization. Ultimately, this process leads to the formation of dental caries (Cheng et al., 2022). Therefore, interventions targeted at the caries-associated virulence factors of *S. mutans*, such as EPS and acid production, as well as acid tolerance, are critical in caries control and prevention.

Given that controlling the primary causative factors is essential for managing chronic diseases, effective daily plaque control plays a critical role in the prevention and management of dental caries (Cheng et al., 2022). Currently, the antibiotic resistance in bacterial biofilms has become a significant public health concern (de la Fuente-Nunez et al., 2023), alternative treatments are urgently needed to address biofilm-driven microbial infections (Tent et al., 2019). Natural products (NPs) have been used as medicines over the past few thousand years. While thousands of NPs have been investigated in medical fields, there are still a large number of NPs with untapped potential in medicine worth exploring. The diverse chemical structures and modes of antimicrobial actions make NPs ideal candidates for natural antimicrobial and anti-caries drugs. Moreover, due to their widespread availability and excellent biosafety profile in the natural world, NPs are increasingly being considered as a promising option for drug discovery and development (Cao et al., 2023). Additionally, NPs can reduce the resistance to conventional antimicrobial drugs through various mechanisms (Porras et al., 2021), providing an approach to overcome the antibiotic resistance.

Isothiocyanates (ITCs) are abundant in cruciferous plants like cauliflower (Vanduchova et al., 2019) with a wide variety of biological effects including anti-inflammatory, anticancer, antibacterial, and antioxidant activities (Li et al., 2022; Treasure et al., 2023; Pogorzelska et al., 2023). Over a hundred natural ITCs have been identified, with the aliphatic compounds being more stable (Fahey et al., 2001). Among various types of ITCs, sulforaphane (SFN, CS(=O)CCCCN=C=S) is the most extensively studied and favored in research related to anticancer, immunomodulation, and cardiovascular and neurological disorders (Elkashty and Tran, 2021; Calabrese and Kozumbo, 2021; Zheng et al., 2022). Besides, SFN exhibited a better safety profile compared to other ITCs, with over a hundred clinical studies conducted on varied SFN-related agents in different fields (Mangla et al., 2021). It has been observed that the potential health benefits of SFN may outweigh the drawbacks, even at high doses (Abdull Razis et al., 2018). Moreover, a wealth of evidence has confirmed the bacteriostatic properties of SFN against a broad spectrum of pathogenic bacteria responsible for disease. It has been widely observed that the extensive pharmacological activity of SFN is intricately linked to its multi-target mode of action (Nowicki et al., 2019). Its antibacterial mechanism primarily involves disrupting the biometabolism and biofilm formation of pathogenic bacteria through modulating specific signaling pathways (Nowicki et al., 2019; Nowicki et al., 2020). Currently, the capability of SFN in combating foodborne pathogens has been thoroughly studied (Krause et al., 2021), while its effects on human infectious agents are still under research (Deramaudt et al., 2020). It is also unknown whether SFN can inhibit the cariogenic virulence of the primary cariogenic bacterium *S. mutans*, and directly contribute to caries prevention. Considering the multiple pharmacological activities and high biosafety of SFN, investigating its potential effects on oral pathogens will lay the foundation for further exploration of its clinical applications.

**Scheme 1:**
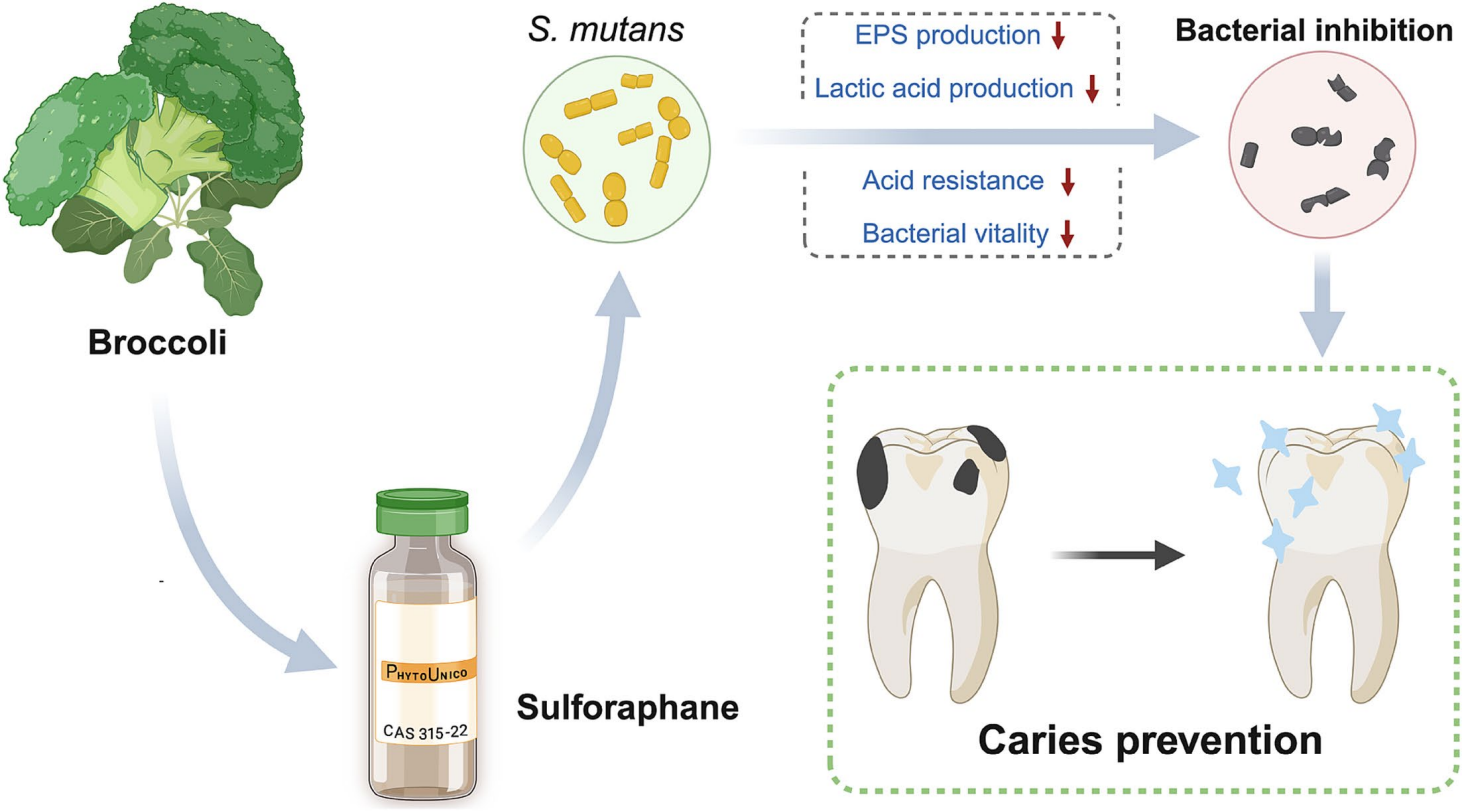
Schematic illustration of SFN in caries control.

The objective of this study was to investigate the antibacterial effects of SFN on both planktonic bacteria and biofilm of *S. mutans*, and to gain deeper insights into how cariogenic virulence factors were affected under SFN application (Scheme 1). For the first time, a comprehensive examination of the impact of SFN on the cariogenic virulence of *S. mutans* was conducted *in vitro*, and the role of SFN in dental caries prevention and control was explored in a rat caries model. The results of this study will provide a robust foundation for the investigation of SFN as a novel natural compound for caries control.

## 2 Materials and methods

### 2.1 Chemicals, bacterial strain and culture conditions

**Figure 1:**
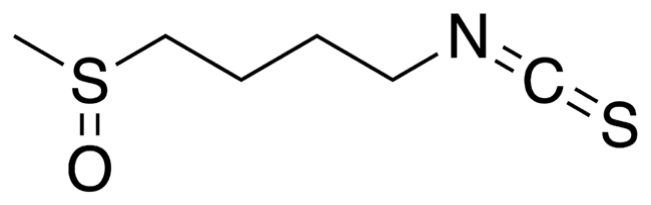
The chemical structure of SFN. Linear form, CS(=O)CCCCN=C=S; molecular weight, 177.3 g/mol.

Sulforaphane (SFN) (≥97% purity) was purchased from Solarbio (Beijing, China) (Figure 1). Reagents included brain heart infusion (BHI) and BHI broth with 1% (w/v) sucrose (BHIS) purchased from Oxoid (UK), chlorhexidine (CHX), dimethyl sulfoxide (DMSO), phosphate-buffered saline (PBS) (pH 7.0) and crystal violet (CV) solution purchased from Sigma (Germany), 3-(4, 5-dimethylthiazol2-yl)-2, 5-diphenyltetrazolium bromide (MTT) and 4% (w/v) paraformaldehyde purchased from Solarbio (Beijing, China), buffered peptone water (BPW) purchased from Haibo (Qingdao, China), and tryptone-yeast extract medium with 20 mM glucose (TYEG) and modified mitis salivarius–bacitracin agar (MSB) purchased from Hopebio (Qingdao, China). The antibacterial activities were evaluated using *Streptococcus mutans* (*S. mutans* UA159) obtained from the American Type Culture Collection (USA). The bacteria were cultured overnight in BHI under anaerobic condition (37°C, 5% CO_2_, 10% H_2_, and 85% N_2_). The concentrations were expressed as weight/volume percentage (w/v) in following assays.

### 2.2 Effect of SFN on *S. mutans in vitro*

#### 2.2.1 Bacterial susceptibility assay

The minimum inhibitory concentration (MIC) of SFN were detected based on the broth microdilution assay (Niu et al., 2020). SFN was dissolved in DMSO at a concentration of 51.2 mg/mL, aliquoted and kept at a temperature of −20°C. 200 μL mixture of different concentrations of SFN (32, 64, 128, 256, 512, 1,024, 2048 μg/mL) and bacterial suspension (1.0 × 10^6^ CFU/mL) was seeded in each microwell. Respective dilutions of 0.5% (w/v) DMSO and CHX with concentration ranging from 1 to 4 μg/mL were tested as controls in this study. The microplate was incubated in anaerobic condition at 37°C for 24 h. The optical density at 600 nm was determined using a microplate spectrophotometer (Multiskan GO, USA). MIC was determined by the lowest concentration of SFN required to inhibit detectable bacterial growth where no apparent growth observed in microwells and OD_600_ values of SFN or CHX present were equal to the blank culture medium. The minimum bactericidal concentration (MBC) was identified by the lowest concentration required to completely inhibit the growth of *S. mutans* on agar plate. The assays were performed in sextuple on six different days to ensure the accuracy of subsequent results.

#### 2.2.2 Kinetic growth curve assay

Bacterial suspensions (1.0 × 10^6^ CFU/mL) were treated with SFN at different concentrations (32, 64, 128, 256 μg/mL) and CHX (2 μg/mL). 0.5% DMSO and culture medium alone were used as the negative control and the blank groups, respectively. The microwell was sealed by 50 μL light mineral oil (Sigma, Germany) to insulate the air and prevent water from evaporating. The microplate was incubated anaerobically at 37°C for 24 h. Absorbances at 600 nm were measured in a microplate spectrophotometer half hourly. The assays were performed in quintuplicate independently.

#### 2.2.3 Crystal violet (CV) staining

900 μL bacterial suspension was added into each well of a 24-well plate in the presence of 100 μL SFN at different concentrations, DMSO or CHX to achieve the final bacterial concentration at 1.0 × 10^6^ CFU/mL. The final concentrations of SFN were sub-MIC levels between 32 and 128 μg/mL with 0.5% DMSO and 1 μg/mL CHX served as controls. After anaerobic incubation for 24 h, the mature biofilm formations were evident at the well bottoms. The supernate was removed and the biofilms were rinsed thrice with PBS. After a 15 min fixation with 4% paraformaldehyde, the 0.1% (w/v) CV solution was applied to stain the biofilms for 5 min. The wells were rinsed gently with distilled deionized water (DDW) and dried before image acquisition though a stereomicroscope (Leica, Germany). 400 μL 95% ethanol was then added into the plate followed by shaking process for 45 min to redissolve the crystal violet dyes. 200 μL mixed liquid was transferred to a 96-well plate to determine the optical density at 595 nm using a spectrophotometer. The ratio of biofilm formation is calculated through the formula:


Biofilm formation=A2/A1×100%

Where A1 is the absorbance of the negative control group at 595 nm and A2 is the absorbances of the experiment groups at 595 nm. The lowest concentration of SFN preventing 90% *S. mutans* biofilm formation in this study was described as the minimum biofilm inhibitory concentration (MBIC). Three replicates were set in the assay and the experiment was repeated five times.

#### 2.2.4 MTT metabolic assay (tetrazolium reduction assay)

Bacteria were cultivated in BHIS for 24 h under the anaerobic conditions at 37°C to form the biofilm, and the mature biofilms were rinsed twice with PBS. Then the biofilms were incubated for another 24 h with increasing final concentrations of SFN (32, 64, 128, 256 μg/mL) and respective concentrations of DMSO (0.5%) and CHX (2 μg/mL). 1 mL MTT (0.5 mg/mL) was introduced to the biofilms and incubated at 37°C for 3 h in complete darkness. After aspirating the supernatant, 1 mL of 100% DMSO was introduced to each well and agitated for 40 min at ambient temperature. Aliquots (200 μL) from the solution were analyzed at 490 nm using a spectrophotometer. Each concentration was determined in three wells and performed for five times.

#### 2.2.5 The colony-forming unit (CFU) counting

Freshly cultured *S. mutans* was diluted to 1.0 × 10^6^ CFU/mL and introduced to a 24-well plate incubated in anaerobic condition at 37°C for 24 h to form biofilms. Supernatant was aspirated and biofilms were rinsed with PBS for three times to wipe off the remained planktonic bacteria. Next, SFN was added to each well to reach the different terminal concentrations ranging from 32 to 256 μg/mL. CHX (2 μg/mL) and 0.5% DMSO were regarded as the positive control and the negative control, respectively. The plate was incubated for another 24 h under the mentioned condition. The treatment of excess medium and biofilms were as mentioned above. The biofilms were harvested and resuspended in PBS following various dilution ranging from 10^4^ -fold to 10^8^ -fold. 100 μL of each dilution were taken and distributed on BHI agar, incubated under an anaerobic environment at 37°C for 48 h. The total colony forming units per well (CFUs) in the original sample were calculated by the following equation:


CFUs=CFU×V×D×10

Where CFU is the countable colony forming units in each agar plate, V is suspension volume, and D is dilution radio. The CFU counting was determined by averaging the number of counted colonies from five plates.

#### 2.2.6 Biofilm morphology observations

For the biofilm formation assay, sterile slides were put in the bottom of 12-well plates. A total of 2 mL mixture of bacterial solution and drugs was introduced to each well, followed by incubated anaerobically at 37°C for 24 h. The concentration of bacteria in the incubation system was 1.0 × 10^6^ CFU/mL, and the terminal concentration of SFN varied from 32 to 256 μg/mL. The control groups consisted of 0.5% DMSO and 2 μg/mL CHX, respectively. Biofilms were gently rinsed with PBS, then fixed with 2.5% glutaraldehyde overnight, prior to gradient dehydration with gradually increasing concentrations of ethanol. The biofilm samples on slides were critically dried, sprayed with gold, and visualized using a scanning electron microscope (SEM) (FEI, Netherlands). For the matured biofilm test, the biofilm was pre-formed on the slide surface as described above, and then incubated with the aforementioned concentrations of SFN for 24 h. The control groups and the subsequent experimental procedures were same as above.

#### 2.2.7 Live/dead staining assays (confocal laser scanning microscopy, CLSM)

Live/Dead Staining Assays was performed using live/dead BacLight bacterial viability kit (L-7012; Molecular Probes, Invitrogen, Carlsbad, CA) to examine bacterial viability and biofilm integrity, as well as to further demonstrate the inhibitory effect of SFN. Live and dead *S. mutans* treated with SFN were labeled using molecular probes SYTO®9 (Invitrogen, USA) and PI (Invitrogen, USA), respectively. Sterile slides were placed in 12-well plates, and the mixed culture solution was introduced following the procedure described in section 2.2.6. Five fields of view were captured for each sample using a CLSM (N-SIM, Nikon, Japan). The software FV31S was applied to visualize the image in different directions. Biomass quantification was assessed by ImageJ COMSTAT (NIH, USA).

#### 2.2.8 Bacteria and extracellular polysaccharide (EPS) staining assay (CLSM)

Bacteria and EPS in the biofilms were stained using SYTO® 9 (Invitrogen, USA) and Alexa Fluor® 647 (Invitrogen, USA) respectively. A negative control was established using 0.5% DMSO. Alexa Fluor® 647 was introduced to label the EPS as the biofilm began to form, while SYTO® 9 was applied to stain the biofilm for 15 min after its formation, with precautions taken to avoid exposure to light. A two-channel scanning observation was carried out using a CLSM (N-SIM, Nikon, Japan), and five fields of view at 60× oil microscope were captured for each sample. Bacteria was viewed in a three-dimensional framework using Imaris 7.0 (Bitplane, Switzerland), and the biomass and distribution of bacterial and EPS were quantified using the image analysis software ImageJ COMSTAT.

#### 2.2.9 Water-insoluble EPS measurement

The biofilm culture method, drug treatments and control group settings were the same as those outlined in section 2.2.4. Planktonic bacteria, water-soluble polysaccharides, and residual medium were removed through rinsing the biofilm with PBS. The biofilms were then collected and resuspended in 1 mL NaOH solution (0.4 M). 200 μL supernatant was collected after centrifugation, mixed with 600 μL anthrone reagent (0.1 g anthrone, 100 mL 98% sulfuric acid) and incubated precisely at 95°C for 6 min. The absorbance at 625 nm was subsequently measured, and the concentration of water-insoluble EPS was calculated using a standard curve. Five replicates were set in the assay.

#### 2.2.10 Glycolytic pH drop assay

Bacteria in the logarithmic growth stage was rinsed with a pre-prepared saline solution (50 mM KCl, 1 mM MgCl_2_) and resuspended in the saline solution containing 1% (w/v) glucose. The pH of the solution adjusted to 7.2 initially. The pH of the solution was then recorded every 15 min using a pH meter (Thermo Scientific, USA) for 2 h. The assay was independently performed five times.

#### 2.2.11 Lactic acid measurement

Biofilms were formed as described in Section 4.2.4 and gently rinsed with PBS. The effect of SFN on acid production was assessed at sub-MIC concentrations (32, 64, 128 μg/mL). 0.5% DMSO and 1 μg/mL CHX were set as controls. Each well was supplemented with 1 mL of BPW medium and anaerobically incubated at 37°C for 2 h. The supernatant was collected after centrifugation (8,000 × g, 5 min, 4°C), and the lactate concentrations were determined using a lactic acid assay kit (Jiancheng, Nanjing, China). The absorbance at 570 nm was then measured and the lactate concentration was calculated based on the standard curve. This assay was performed in quintuplicate on three different occasions.

#### 2.2.12 Acid tolerance test

Bacteria cultured to mid-logarithmic stage were collected and resuspended in TYEG, with pH adjusted to 5.0 using phosphate citrate buffer (Li et al., 2020). The bacterial concentration was standardized to 1.0 × 10^6^ CFU/mL and treated with varying concentrations of sub-MIC SFN from 32 to 128 μg/mL before anaerobically incubating at 37°C for 2 h. Bacterial suspensions treated with 0.5% DMSO and 1 μg/mL CHX were applied as negative and positive controls, respectively. Samples were obtained before and after treatment for viable bacterial CFU counting. The experiment was repeated in quintuplicate.

#### 2.2.13 RNA extraction and quantitative RT-PCR

Bacterial suspensions were treated with SFN at different sub-inhibitory concentrations as mentioned above and cultured to form biofilm. 0.5% DMSO was used as the negative control group. The biofilms were disintegrated using sonication, and subsequently, the dispersed biofilm samples were collected through centrifugation. MasterPure™ RNA Purifcation Kit (Lucigen Wisconsin, USA) was used to extract and purify total RNA in compliance with the manufacturer’s instructions. The quantity and purity of RNA were determined using a Nanodrop 2000 spectrophotometer. For cDNA synthesis, the PrimeScript™ RT Reagent Kit with gDNA eraser (TAKARA BIO INC, Shiga, Japan) was employed to make sure no contamination. The specific genes tested and the primer sequences are listed in Table 1. Subsequently, qRT-PCR was conducted on a LightCycler® 480 System (Roche, Switzerland) following the instructions of the TB GreenTM® Premix Ex Taq™ II Kit (RR820A; Takara). The two-step protocol involved denaturation step at 95°C for 15 s, followed by 40 cycles of denaturation at 60°C for 30 s per cycle. To determine the relative expression levels of the different genes, the data was normalized against the 16S rRNA gene transcripts using the 2^−ΔΔCT^ method. Five replicates were set in the assay.

**Table 1:** Specific primers of *S. mutans* used for qRT-PCR.

Gene	Forward primer	Reverse primer	Reference
16S rRNA	AGCGTTGTCCGGATTTATTG	CTACGCATTTCACCGCTACA	Wu et al. (2022)
*gtfB*	TACACTTTCGGGTGGCTTGG	AGAAGCTGTTTCCCCAACAGT	Wu et al. (2022)
*gtfC*	AGCAGATTCAACTGACGACCG	TCAGTAACAGTGGCGGTTGG	Wu et al. (2022)
*gtfD*	TGCAAGCGACGGAAAACAAG	GCCTGTCAGAGCTTCACCAT	Wu et al. (2022)
*ldh*	AAAAACCAGGCGAAACTCGC	CTGAACGCGCATCAACATCA	Sun et al. (2014)
*atpD*	TGTTGATGGTCTGGGTGAAA	TTTGACGGTCTCCGATAACC	Wang et al. (2019)
*vicR*	CGCAGTGGCTGAGGAAAATG	ACCTGTGTGTGTCGCTAAGTGATG	Wang et al. (2019)
*comC*	GACTTTAAAGAAATTAAGACTG	CTCTGATTGACCATTCTTCTGG	Li et al. (2018)
*comD*	CTCTGATTGACCATTCTTCTGG	CATTCTGAGTTTATGCCCCTC	Li et al. (2018)
*comE*	CCTGAAAAGGGCAATCACCAG	GGGGCATAAACTCAGAATGTGTCG	Li et al. (2018)

### 2.3 Effects of SFN on dental caries development *in vivo*

#### 2.3.1 Rat caries model

Animal experiments were performed on a modified animal model of caries (Hajishengallis, 2023), approved by the West China Dental Ethics Committee (WCHSIRB-D-2022-520). The environment and conditions of the facility were in accordance with GB14925-2010 and ARRIVE guideline 2.0 (Percie et al., 2020). A total of forty 17-day-old male specific-pathogen-free (SPF) SD rats were procured from the Animal Experiment Center of Sichuan University. To suppress the primary flora in their oral cavities and facilitate *S. mutans* colonization, the rats were fed with specific feed (0.1% chloramphenicol, 0.1% ampicillin, and 0.1% carbenicillin), along with drinking water containing 400 kU penicillin for 3 days (Yucesoy et al., 2018; Wang et al., 2018). The oral colonization in the rat model has been assessed prior to the inoculation with *S. mutans* using Mitis-Salivarius-Bacitracin (MSB) plates. The cariogenic diet (2000#; 56% sucrose; Trophic, Nantong, China) was introduced, and *S. mutans* bacterial solution (10^8^ CFU/mL) in the logarithmic growth phase was applied to the intraoral and dental surfaces of rats using a cotton swab for three consecutive days. After confirming *S. mutans* colonization through MSB plates, rats were allocated into 4 groups (*n* = 10) through a random process: positive control group (NaF, 1,000 ppm F^−^), negative control group (0.5% DMSO), and two experimental groups (256 and 512 μg/mL SFN). A small cotton swab with a diameter of 1 mm (Kangjie Medical, Putian, China) soaked with the respective treatment solutions was used to brush the tooth surface and oral mucosa of the rats three times a day, with each session lasting 1 min, for four consecutive weeks. The body weight and health condition of the rats were monitored on a weekly basis, and euthanasia was performed by CO_2_ asphyxiation after 4 weeks. The mandible and maxilla were aseptically dissected, and the tongue, palate, buccal mucosa, as well as organs such as the heart, liver, and kidneys, were excised for subsequent experiments.

#### 2.3.2 Micro-CT analysis

Micro-computed tomography (micro-CT) (μCT50; SCANCO, Switzerland) was used for qualitative and quantitative analysis of the tooth structure and density with high accuracy (Alqareer et al., 2022). In this study, the mandibles were scanned with micro-CT at the resolution of 500 proj/180° and scanning accuracy of 10 μm. The mandibles were then reconstructed, followed by bone mineral density (BMD) and bone volume per tissue volume (BV/TV) calculation.

#### 2.3.3 Keyes score assessment

The surrounding tissue was carefully debrided as much as possible to separate the teeth without causing damage to tooth structures. Subsequently, all jaws were immersed in a 4% paraformaldehyde solution for fixation. Afterwards, they were stained with 0.4% ammonium purpurate solution for 12 h. The pits and fissures, and smooth surfaces of all teeth exposed after sagittal hemisection were successively photographed using a body microscope (Leica, Germany). To evaluate the extent of caries, the smooth surfaces and the fossae were scored according to the Keyes scoring criteria (Keyes, 1958).

#### 2.3.4 *In vivo* biological safety evaluation

Histopathological evaluation of the tongue, palate, buccal mucosa, as well as organs including the heart, liver, and kidneys, was conducted using hematoxylin–eosin (H&E) staining (Jiang et al., 2022). The specimens were fixed with a 4% paraformaldehyde solution for 24 h, sectioned and dehydrated, and subsequently stained with hematoxylin–eosin. Digital section scanning was performed using a scanning system (PRECICE, Beijing, China), and images were captured and analyzed using CaseViewer 2.1 software (3DHISTECH, Hungary).

### 2.4 Statistical analysis

The SPSS 26.0 (IBM, Chicago, IL, USA) and GraphPad Prism 9 (GraphPad Software Inc., CA, USA) were used to carry out the statistical analysis. One-way analysis of variance (ANOVA) was performed to compare the difference among groups, followed by Tukey’s honestly significant difference (HSD) test. Images of SEM and CLSM were reconstructed and analyzed using Imaris and Image J. The level of significance was set at 5% (*p* < 0.05).

## 3 Results

### 3.1 Inhibition on growth of *S. mutans* plankton

**Figure 2:**
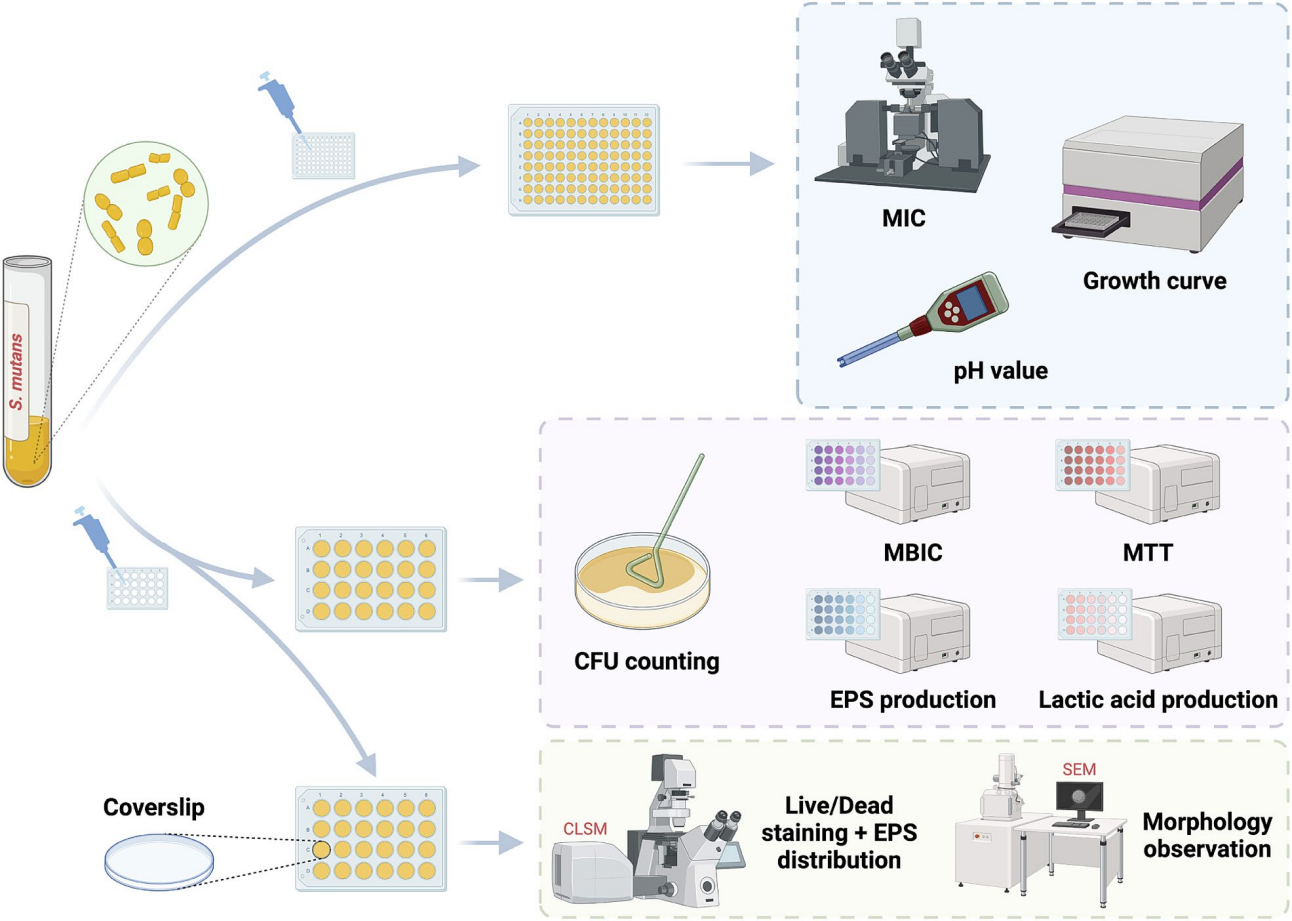
The experiment procedure *in vitro*.

**Figure 3:**
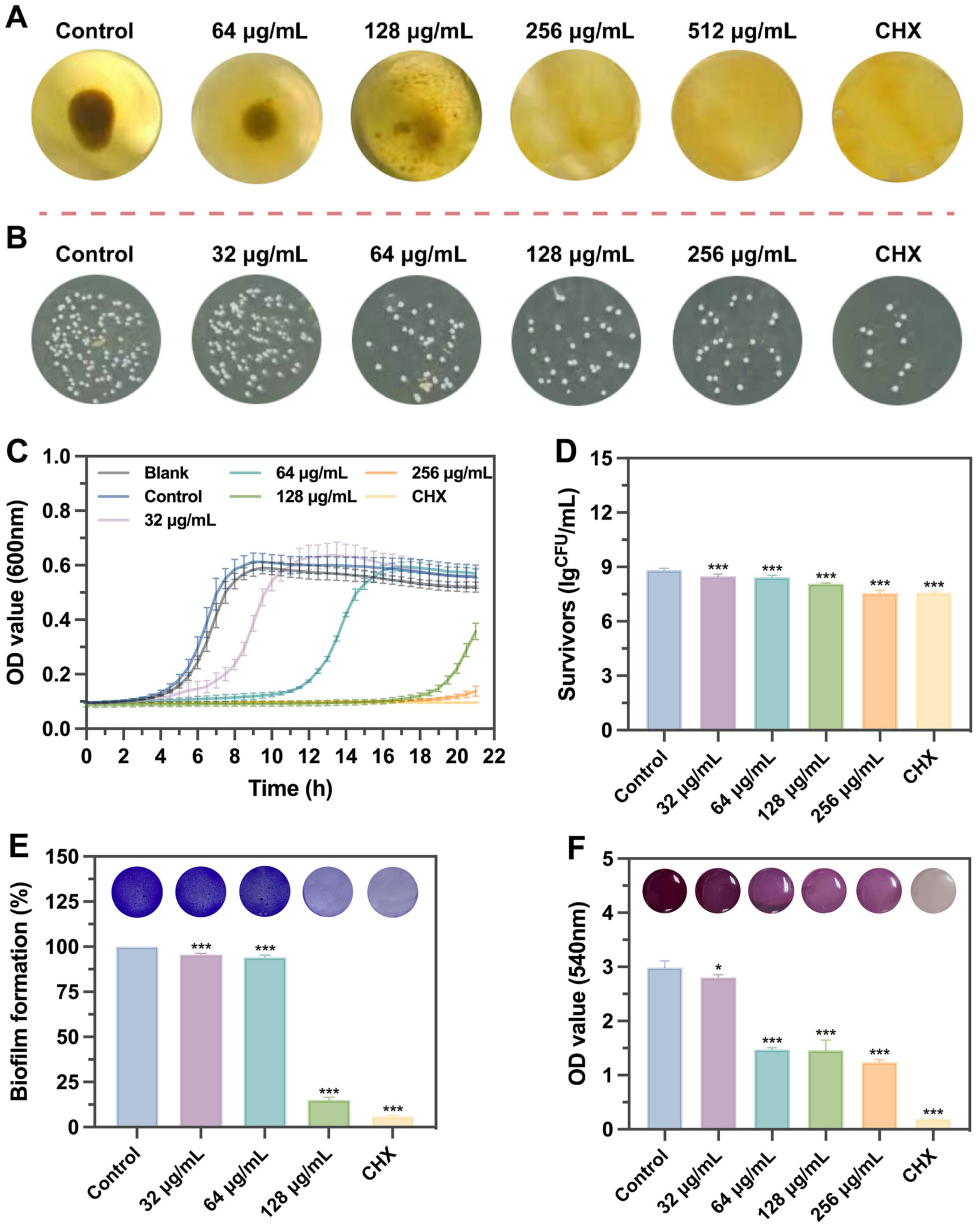
Effects of SFN on the growth of *S. mutans* planktonic bacteria and biofilm formation. **(A)** Observation of bacterial growth in U-bottomed microtiter wells by broth dilution method. **(B)** Representative bacterial colony images of different groups untreated or exposed to chemicals. **(C)** A typical kinetic growth curves under the treatment of SFN. **(D)** CFUs counting after biofilm exposure to different concentrations of SFN or CHX. **(E)** The effect of SFN on *S. mutans* biofilm formation evaluated using CV staining. Typical pictures of stained biofilms are presented above the bars. **(F)** The cell viability of *S. mutans* within preformed biofilms after the incubation with SFN for 24 h. Above the bars are different colors presented in the solution after metabolism of MTT by different groups of viable cells. All values represent the means ± standard deviation (*n* = 5) (*p < 0.05, ***p < 0.001, compared to the negative control group).

*S. mutans* UA159 was used to evaluate the biological functions of SFN *in vitro* (Figure 2). Initially, we treated *S. mutans* with various concentrations of SFN to identify the inhibitory effect of SFN on the floating bacteria (Figure 3). The minimum inhibition concentration (MIC) of SFN tested on planktonic *S. mutans* was 256 μg/mL (Table 2), which was visually displayed in Figure 3A, while the minimum bactericidal concentration (MBC) was not detected in the involved concentration range. CHX was used as the positive control with the MIC value being 2 μg/mL and the MBC value being 4 μg/mL. No effect was observed on bacterial growth with the existence of 0.5% DMSO as the solvent control.

**Table 2:** The minimum inhibition concentration (MIC), minimum bactericidal concentration (MBC) and minimum biofilm inhibitory concentrations (MBIC) against *S. mutans*.

Bacterial strain	SFN (μg/mL)			CHX (μg/mL)	
	MIC	MBC	MBIC_90_^1^	MIC	MBC
*S. mutans* UA159	256	>1,024	128	2	4

^1The lowest concentration of SFN preventing 90% S. mutans biofilm formation.^

Kinetic growth curves were further conducted to access the inhibitory effects of SFN on the growth of *S. mutans* (Figure 3C). We found SFN exhibited a potently antibacterial properties against *S. mutans* at sub-MIC levels. A prolonged growth lag phase was exhibited when *S. mutans* was exposed to increasing concentrations of SFN below the MIC, which was particularly obvious in the 1/2 MIC (128 μg/mL) group. The OD values of the group treated with 256 μg/mL SFN were essentially the same as those of the positive control group, suggesting that bacterial growth was completely suppressed by this concentration of SFN throughout the entire assay duration. This outcome indicated that SFN did not exhibit bactericidal properties against *S. mutans*; however, it did possess a potent inhibitory effect.

### 3.2 Inhibition on biofilm formation and metabolic activity of *S. mutans*

SFN showed strong inhibitory effects on the formation of *S. mutans* biofilm in a dose-dependent manner. The *S. mutans* biofilms exposed to SFN were quantified by colony counting (Figure 3B). The overall biomass in *S. mutans* biofilm was markedly reduced by 32 μg/mL SFN, and the reduction of viable colony forming units (CFUs) in biofilm were even more significant with increasing concentration (Figure 3D). SFN inhibited the biomass and metabolic activities in the formed biofilm. The addition of SFN at sub-MIC level to *S. mutans* culture effectively reduced biofilm formation. The result of crystal violet (CV) staining revealed a significant reduction in overall biomass of *S. mutans* biofilm, particularly evident in the treatment group exposed to the concentration of 128 μg/mL (Figure 3E). Living cells metabolize specific yellow reagent (MTT) to blue-violet formazan, and changes in the metabolic capacity of *S. mutans* biofilms can be assessed using the MTT assay (Ghasemi et al., 2021). Low concentrations (64 μg/mL) of SFN significantly decreased the metabolic activity of *S. mutans* biofilm as illustrated in Figure 3F.

**Figure 4:**
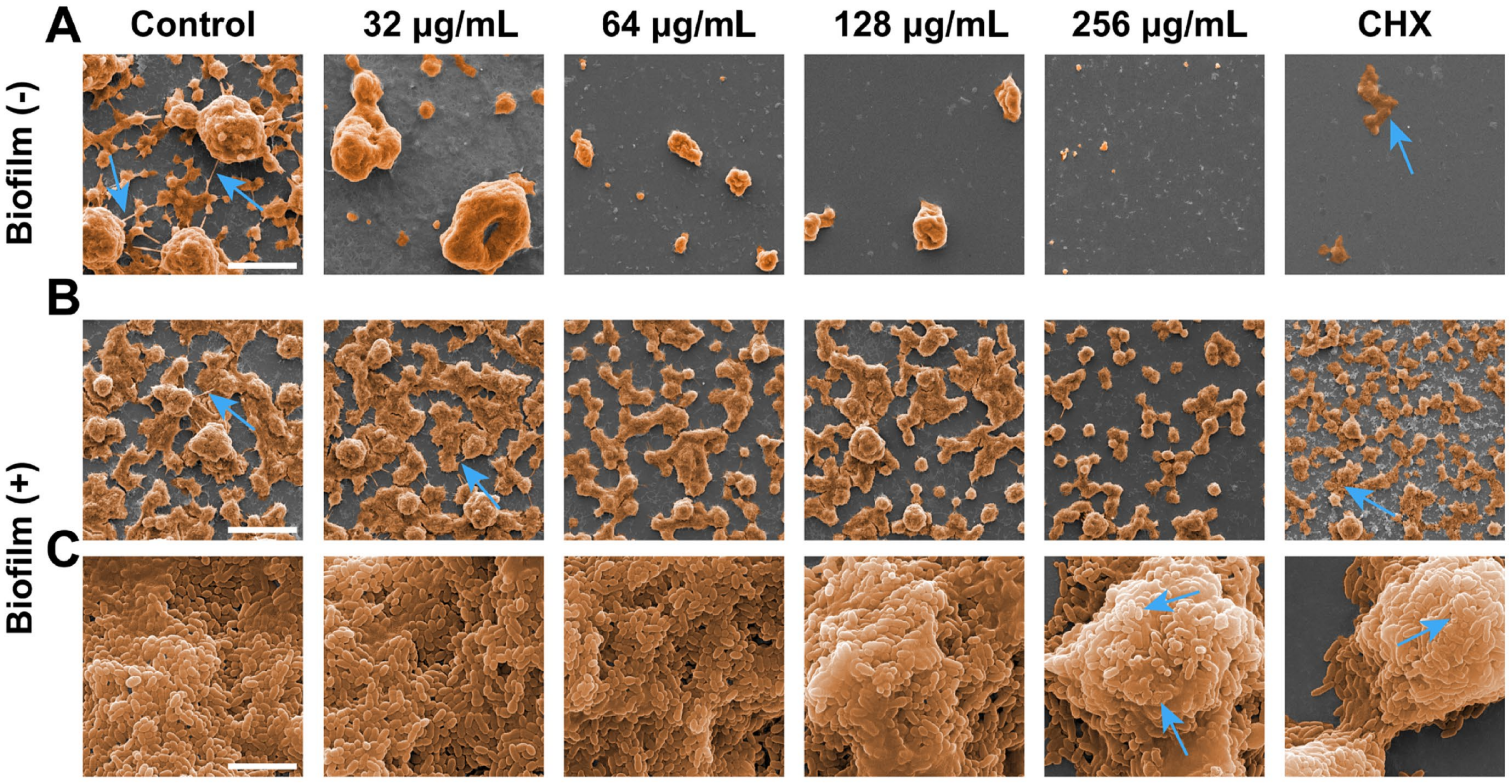
Inhibitory and destructive effects on *S. mutans* biofilms. **(A)** SEM images of *S. mutans* biofilms formation on coverslips captured at a magnification of 1,000× (scale bar: 10 μm). SEM images acquired at two different magnifications: **(B)** 1,000× (scale bar: 10 μm) and **(C)** 20,000× (scale bar: 200 μm) after the *S. mutans* biofilms exposed to various chemicals for 24 h. Blue arrows represent the inter-colony connection and extracellular matrix produced by *S. mutans*.

**Figure 5:**
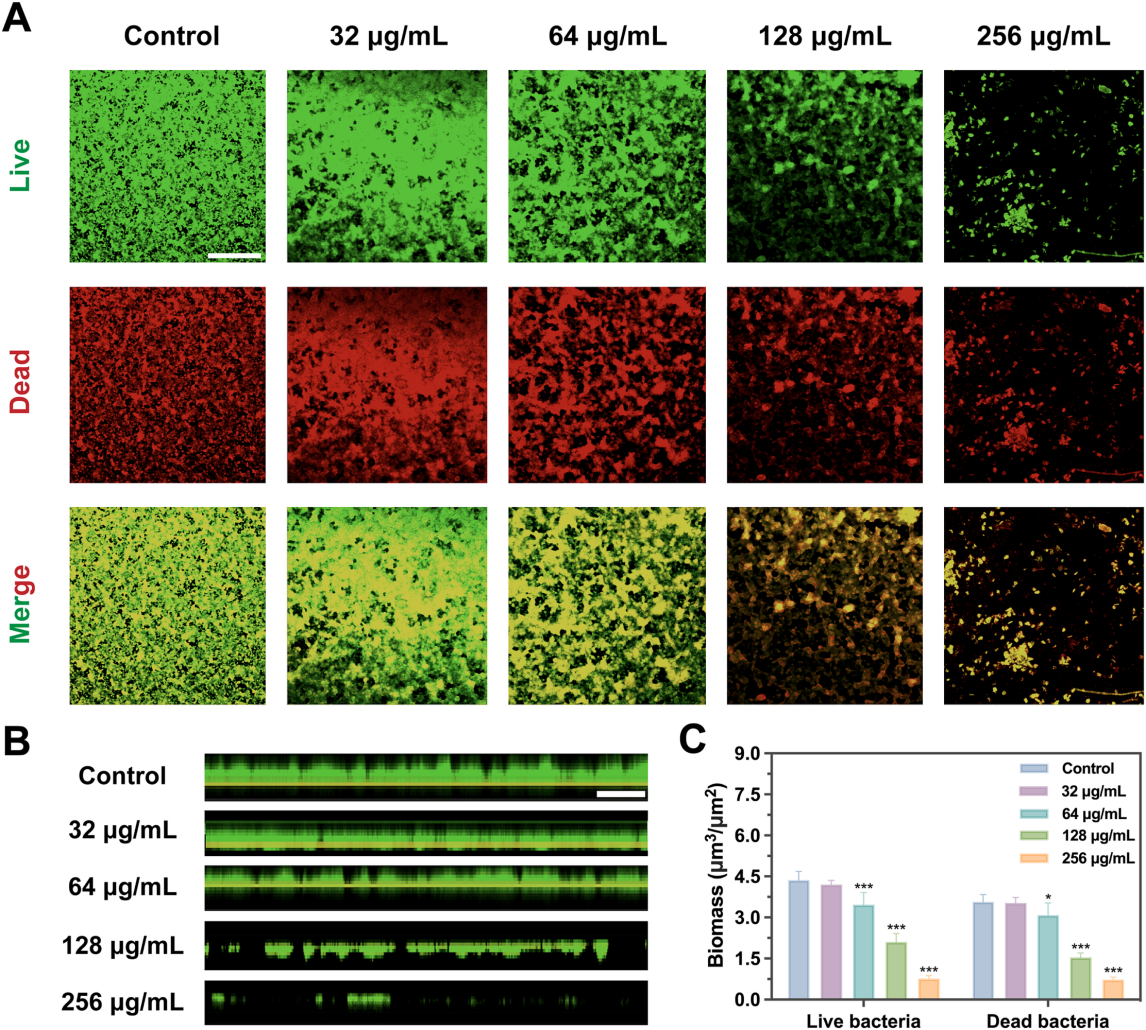
Bacterial viability in *S. mutans* biofilms with SFN treatment based on CLSM. **(A)** Representative images of live/dead staining of *S. mutans* biofilm after treatment with SFN (scale bar: 500 μm). **(B)** Images of stained biofilms with SFN treatment obtained by CLSM (scale bar: 500 μm). The green color (SYTO-9) indicates live bacteria. **(C)** Evaluation of live and dead bacterial biomass in *S. mutans* biofilms based on analysis of five randomly selected fields using COMSTAT. (*p < 0.05, ***p < 0.001, compared to the negative control group).

**Figure 6:**
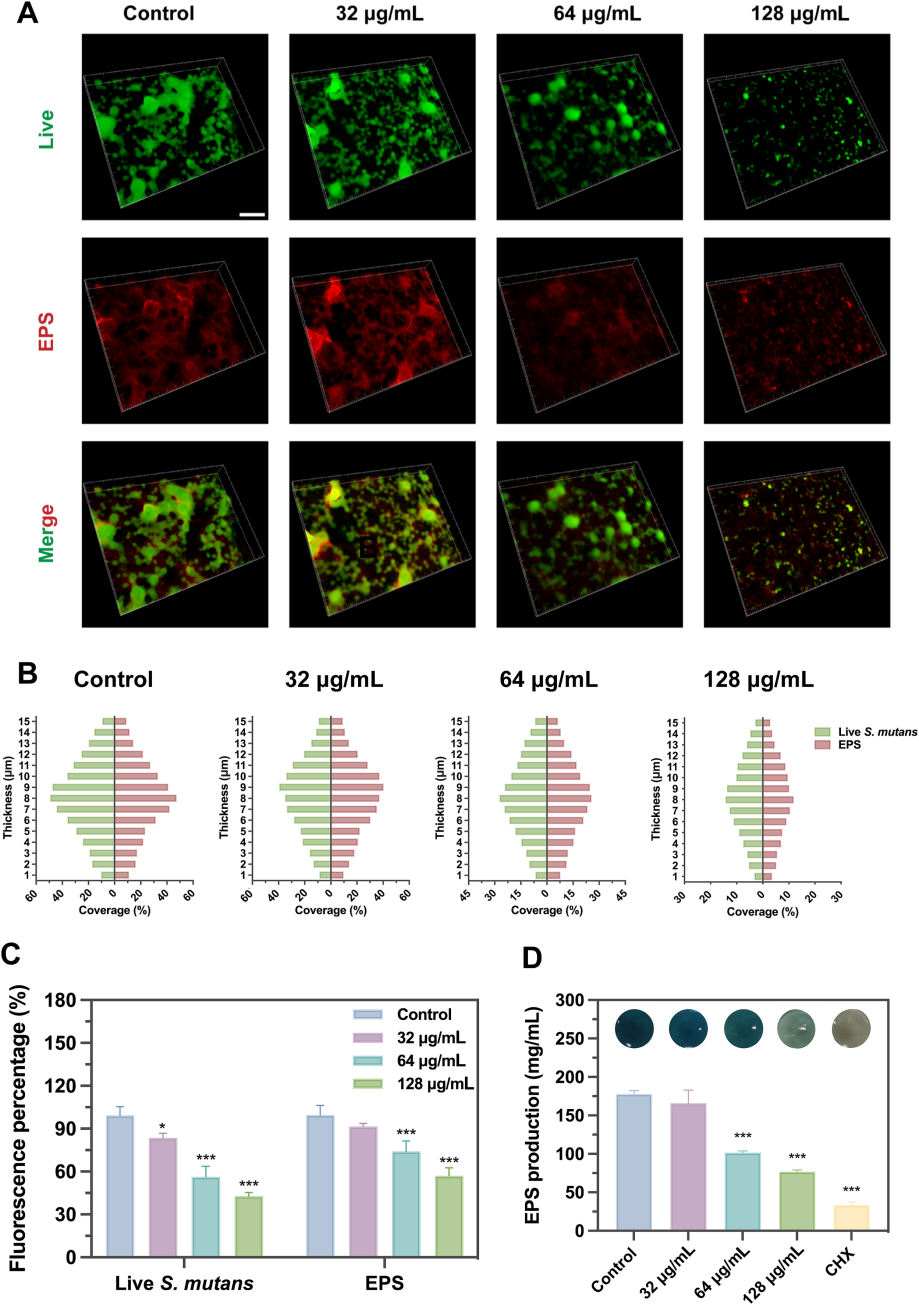
Effects of SFN on EPS and biofilm formation. **(A)** Fluorescent images of *S. mutans* biofilms treated with SFN at sub-MIC levels captured by CLSM (scale bar: 200 μm). Bacterial cells are shown in green and EPS in red. **(B)** The distribution of bacteria and EPS across scanned layer of *S. mutans* biofilms, calculated by Image J COMSTAT. **(C)** Fluorescence intensity of SYTO® 9 and Alexa Fluor® 647, respectively, applied to label live cells and EPS. **(D)** Effects of SFN on synthesis of EPS at sub-inhibitory concentrations. Above the bars are different colors presented in the solution after reaction. All values represent the mean ± standard deviation (*n* = 5) (*p < 0.05, ***p < 0.001, compared to the negative control group).

Scanning electron microscope (SEM) and confocal laser scanning microscope (CLSM) results visualized the inhibitory effect of SFN on *S. mutans* biofilm. The observations of SEM and CLSM revealed a reduction in *S. mutans* bacteria aggregation and biofilm formation on the slides (Figures 4–6) (Supplementary material). After 24 h of the treatment, *S. mutans* formed a thin biofilm on the surface of slides, with its biomass inversely proportional to the concentration of SFN (Figure 4). As shown in Figure 5, the 256 μg/mL SFN treatment of *S. mutans* was not fully effective in eliminating the cells, as a small amount of live bacteria and biofilms remained visible. CLSM results revealed that the green fluorescence intensity in the negative control group was notably higher, with the biofilm displaying an overall green appearance. In contrast, the SFN-treated group exhibited weaker green fluorescence, and the 128 μg/mL SFN-treated group displayed scattered green fluorescence (Figures 5A, 6A). Quantitative fluorescence analysis and live/dead bacteria biomass analysis indicated a significant reduction in viable bacterial biomass in the 64 and 128 μg/mL SFN treatment groups (Figures 5C, 6C). Meanwhile, biofilm thickness was reduced after SFN treatment. As the concentration of SFN increased, the biofilm became thinner and its integrity was disrupted (Figures 5B, 6B). Overall, these results demonstrated that SFN effectively suppressed the formation and biomass of *S. mutans* biofilm.

### 3.3 Destructive effects on *S. mutans* biomembrane structure

Given the significant effect of SFN on inhibiting *S. mutans* biofilm biomass, we employed SEM to access the impact of SFN on the surface morphology and microstructure of *S. mutans* biofilm. As illustrated in Figure 4, the negative control group exhibited a dense biofilm on the slide surface visible at low magnification (1000×), with a complex biofilm structure consisting of a dense extracellular matrix and microspherical biofilm clusters (Figures 4A,B). At high magnification (20,000×), the dense arrangement of bacterial organisms within the biofilm was visible (Figure 4C). Following treatments with 64, 128, and 256 μg/mL SFN, the biofilm collapsed, and the patchy biofilm on the slide surface reduced significantly compared with the negative control group at the low magnification, with microspherical colonies still visible. The biofilm structure in the SFN treatment group became looser compared to that in the negative control group at a high magnification. Treatments with 128 and 256 μg/mL SFN made it difficult to form a complete biofilm structure. The microglobular biofilm structure nearly disappeared under the 1,000× microscope, with a significant reduction in biofilm matrix and bacteria (Figure 4B). *S. mutans* treated with CHX failed to form biofilm, exhibiting protein-like patchy precipitation under the microscope (Figures 4A,B). The impact of SFN on the micro-morphology and structure of preformed biofilm was not as significant as that of CHX, with the SFN treatment resulting in a certain biofilm structure, but a significantly reduced extracellular matrix and biofilm plaque. Microscopic imaging of the biofilm structure revealed that SFN caused a dose-dependent degree of damage to *S. mutans* and the biofilm structure, with the 256 μg/mL SFN-treated group exhibiting collapsed bacterial bodies and the disrupted cell membrane surface structure under the high magnification microscope.

### 3.4 Reduction in the synthesis of EPS

Treatments with SFN at the sub-MIC levels exhibited a significant inhibitory effect on the EPS synthesis of *S. mutans*. The distribution of EPS labeled with red color in *S. mutans* biofilm was observed under CLSM (Figure 6A). Treatments with 64 and 128 μg/mL SFN resulted in a substantial decrease in the quantity of EPS synthesized as well as the overall coverage and distribution of EPS (Figures 6B,C). Notably, there was a clear dose-dependent reduction in EPS synthesis. The anthrone-sulfuric acid method was used to confirm the inhibitory effect of SFN on *S. mutans* EPS synthesis (Figure 6D).

### 3.5 Reduction in lactic acid production and acidurance

**Figure 7:**
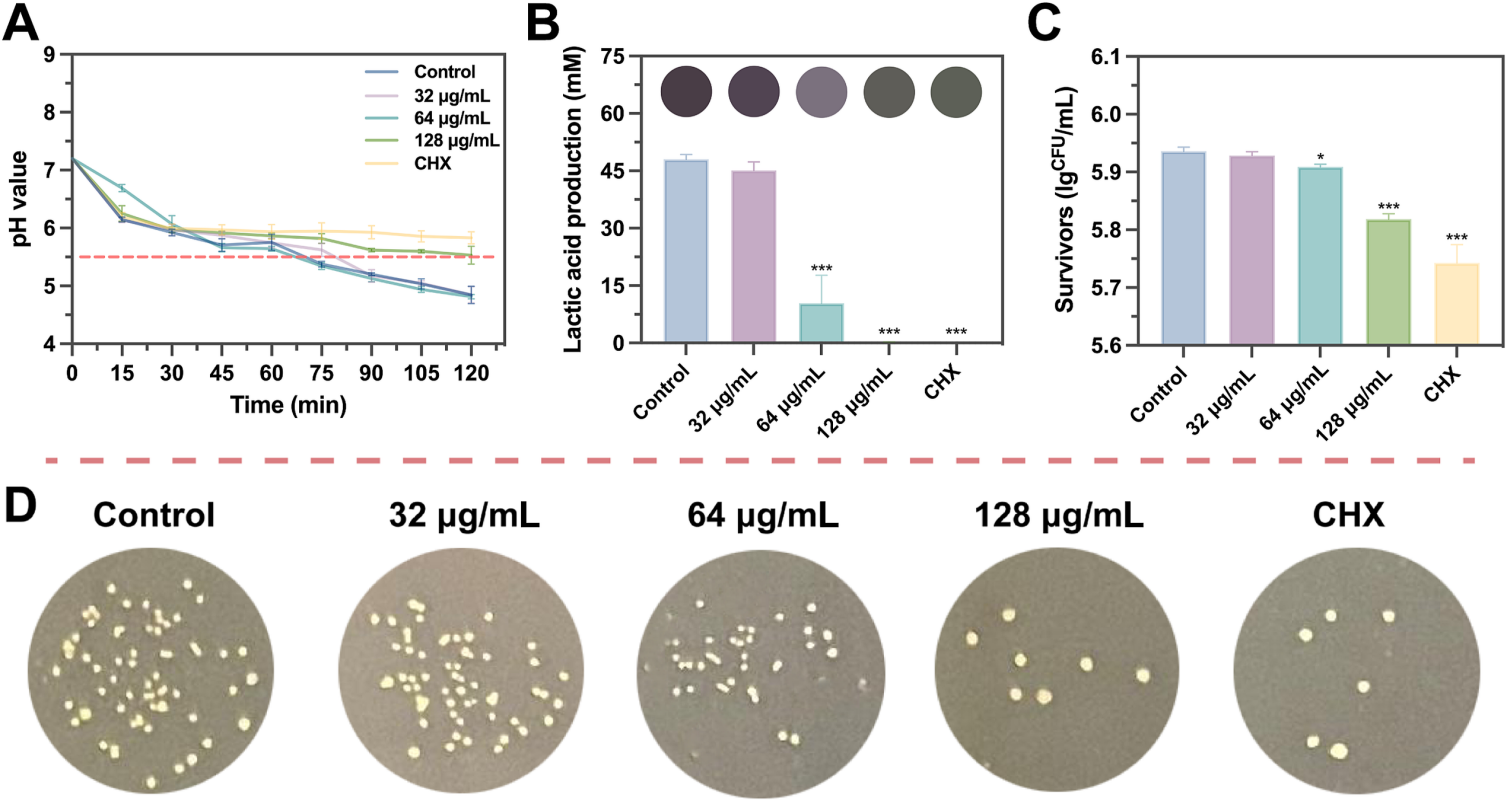
Inhibition of SFN on *S. mutans* lactic acid production and acidurance. **(A)** The pH value of the bacterial suspension was accessed at different incubation times. **(B)** Effects of SFN on the production of lactic acid by *S. mutans* biofilm at sub-inhibitory concentration. **(C)** Acid tolerance of *S. mutans* following treated with SFN and Control reagents in an acid environment (pH 5.0). **(D)** Representative bacterial colony images of different groups in a lethal acid environment with pH equal to 5.0. CHX was used as a positive control at the concentrations of 1 μg/mL. The results presented are expressed as the mean ± SD of three independent experiments. The critical pH level leading to demineralization is indicated by a dashed line. All values represent the mean ± standard deviation (*n* = 5) (*p < 0.05, ***p < 0.001, compared to the negative control group).

The effect of SFN at sub-inhibitory concentrations on *S. mutans* acid production was investigated though measuring lactate production during glycolysis and the resulting decrease in pH of the culture medium. As depicted in Figure 7A, treatment with 128 μg/mL SFN considerably slowed down the rate of acid production, resulting in a terminal pH of the medium close to the critical demineralization threshold (pH 5.5) after 2 h. Nevertheless, the terminal pH in the SFN-treated group remained significantly elevated in comparison with the negative control group. Moreover, the roles of 32 and 64 μg/mL SFN on the decline of medium pH were less pronounced. Quantitative analysis of lactic acid production demonstrated that 64 μg/mL SFN significantly inhibited the properties of acid generation by *S. mutans*, whereas lactic acid was nearly undetectable in the 128 μg/mL SFN and CHX-treated groups (Figure 7B).

To further assess the impact of SFN on the aciduric property of *S. mutans*, the survival rate of bacteria was evaluated following SFN treatment and exposure to the environment of pH at 5.0 for 2 h (Figure 7C). As shown in Figures 7C,D, 128 μg/mL SFN significantly reduced the proportion of viable bacteria in the pH 5.0 environment. Collectively, these findings indicated that SFN inhibited the acid production, a key cariogenic virulence factor responsible for tooth demineralization, as well as the capacity of *S. mutans* to survive in an acidic environment.

### 3.6 Downregulation in genes related cariogenic virulence and quorum sensing (QS)

**Figure 8:**
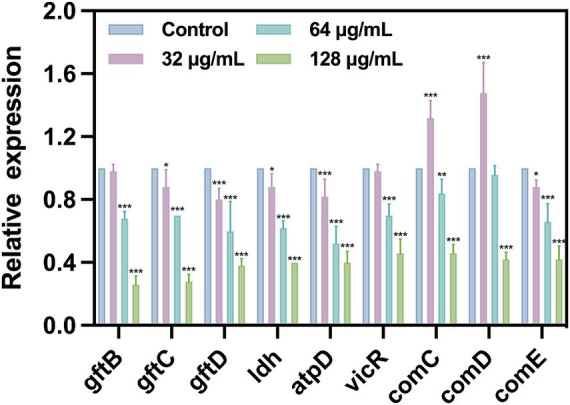
Expression of specific gene in *S. mutans* after treated with SFN at sub-inhibitory concentration. All values represent the mean ± standard deviation (*n* = 5) (*p < 0.05, **p < 0.01, ***p < 0.001, compared to the negative control group).

To further investigate the inhibitory effect of SFN on *S. mutans*, we evaluated the impact of SFN at sub-MIC levels on the expression of genes related to cariogenic virulence factors and QS by using qRT-PCR (Figure 8). Our results revealed that the expression of genes related to EPS synthesis (*gtfB*, *gtfC*, and *gtfD*), acid production (*ldh*), and acidurance (*atpD*) were downregulated upon treatment with SFN, which was consistent with the outcomes of the phenotyping experiments *in vitro*. The expression of *gtfC*, *gtfD*, *ldh*, and *atpD* was significantly down-regulated after SFN treatment at a concentration of 32 μg/mL, except for *gtfB*. Interestingly, this concentration of SFN significantly upregulated the expression of *comC* and *comD*, which are closely associated with QS. Conversely, 64 μg/mL SFN exhibited a notable inhibitory effect on the expression of virulence genes *gtfB*, *gtfC*, *gtfD*, and *ldh*, while downregulating the expression of *comC and comE* genes. However, no significant effect was observed on the expression of *comD* genes. Furthermore, SFN significantly inhibited the expression of the *vicR* gene in a dose-dependent manner, which plays a crucial role in adapting to environmental changes. At a concentration of 128 μg/mL, SFN inhibited the expression of all genes in *S. mutans*, exhibiting the most significant effect. Collectively, our findings demonstrated that SFN at sub-inhibitory concentrations exerted a transcriptional downregulation of genes associated with cariogenic virulence in *S. mutans*.

### 3.7 *In vivo* anti-caries efficacy

**Figure 9:**
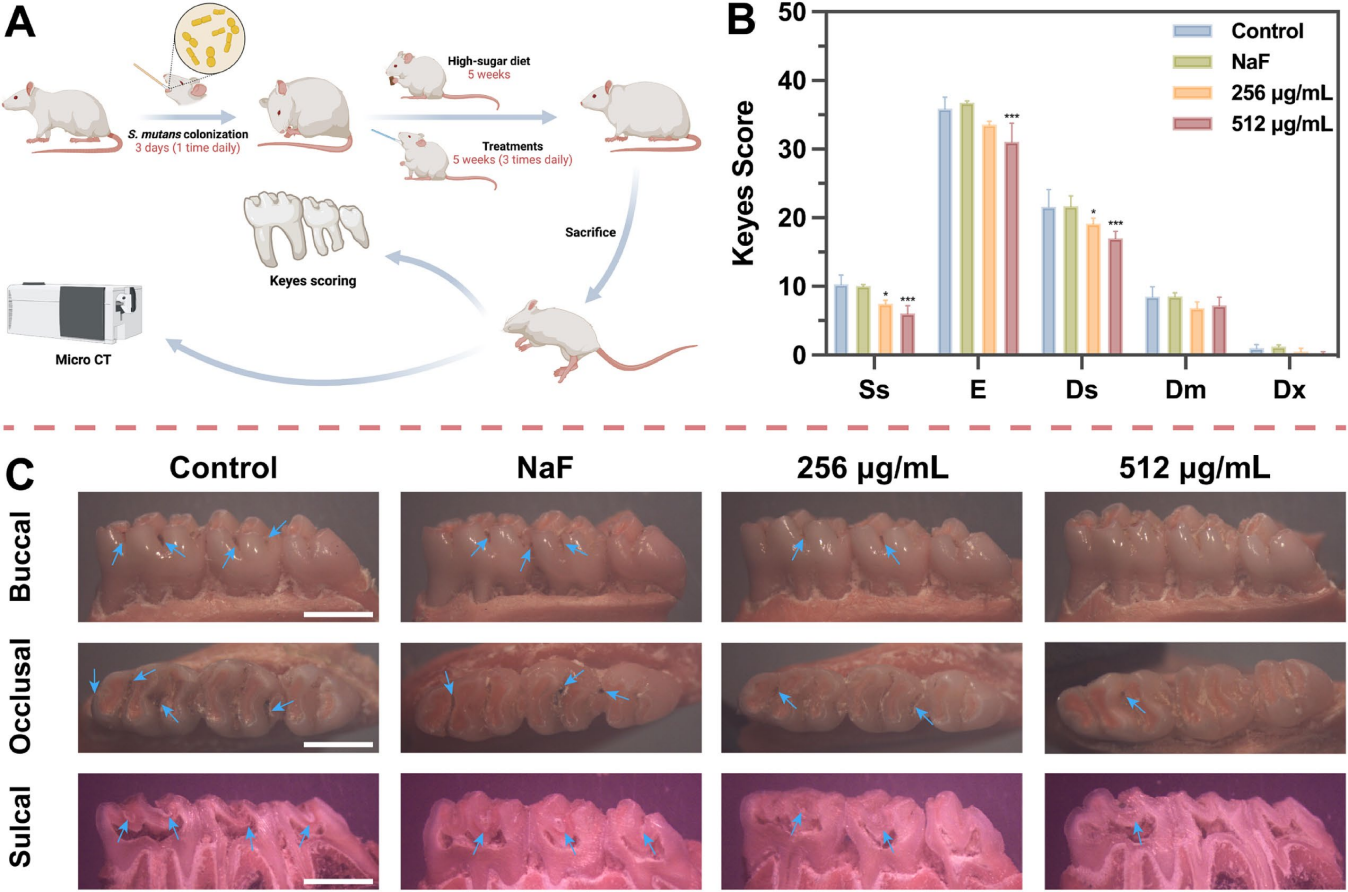
The role of SFN on dental caries *in vivo* study. **(A)** The experiment procedure in vivo. **(B)** Caries scores were used to evaluate both stages and extent of the severity in caries. Ss means smooth surface lesion. Sulcal surface lesions were categorized and graded into four levels: confined to enamel (E), confined to 1/4 of dentin (Ds), confined to 3/4 of dentin (Dm) and all dentin involved (Dx). **(C)** Photographs of Keyes scores staining. Blue arrows indicate areas of caries. (Scale bar: 500 μm). All values represent the mean ± standard deviation (*n* = 10) (*p < 0.05, ***p < 0.001, compared to the negative control group).

The direct impact of SFN on caries development was further evaluated by establishing a rat caries model (Figure 9A). As depicted in Figure 9C, caries lesions were stained red using ammonium purpurate solution, with more prominent caries observed on the smooth surface and in the fossa of the control group. In the NaF-treated group, the demineralized area stained red beneath the mineralized layer remained visible in the sagittal section (Figure 9C). There was no significant difference observed in the depth of caries between NaF-treated group and the negative control group (Figure 9B), possibly due to incomplete formation or mineralization of the remineralized layer. However, the topical treatment with SFN notably decreased the incidence and severity of fossa caries, particularly at a concentration of 512 μg/mL (Figure 9B).

**Figure 10:**
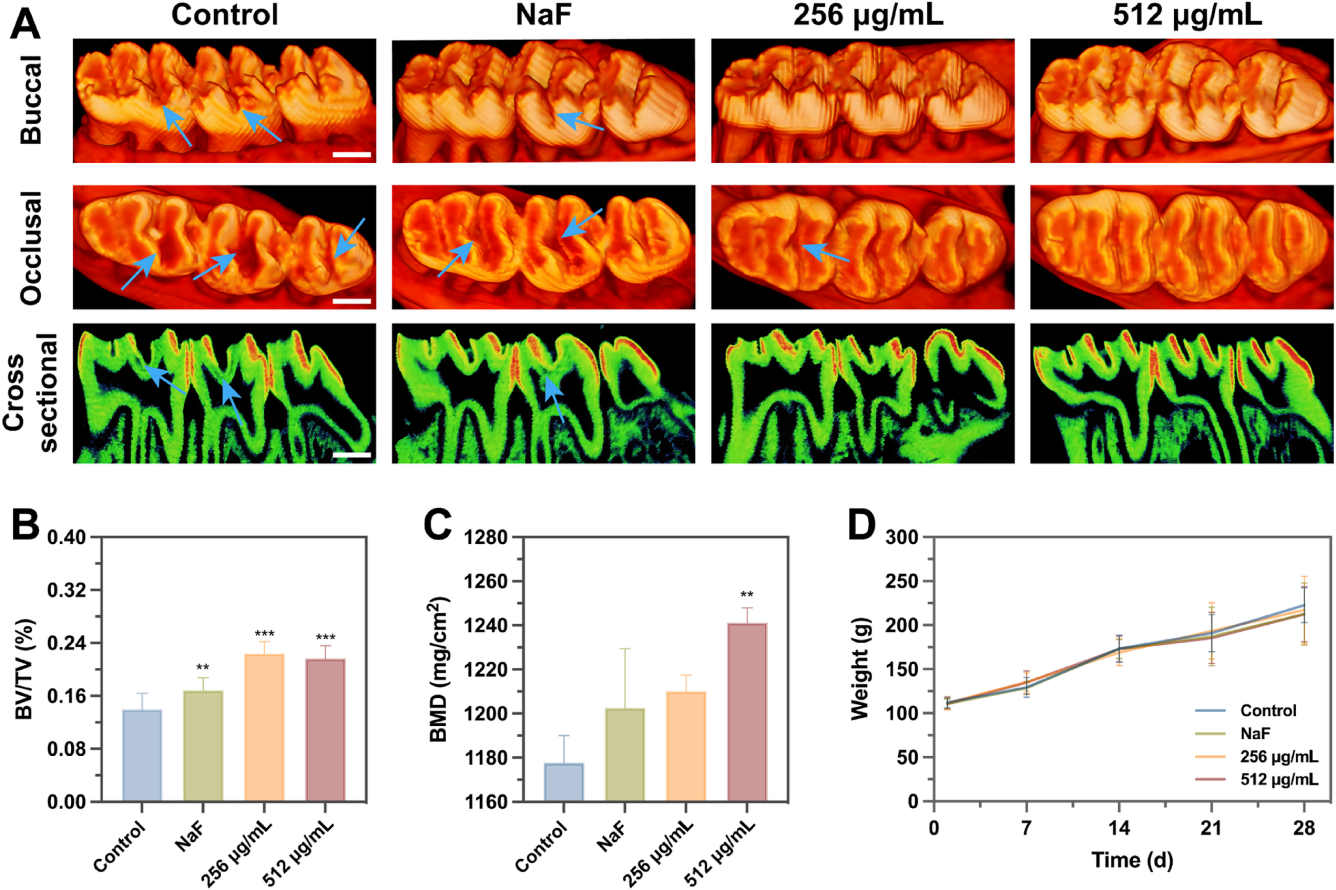
Effects of SFN on microarchitecture of molars and weight. **(A)** Three-dimensional and sagittal images of molar areas. (Scale bar: 500 μm). **(B)** BV/TV and **(C)** BMD were quantitatively measured. **(D)** The body weight of rats was recorded weekly to monitor the local and systematic influence of SFN on their health throughout the duration of the experiment. All values represent the mean ± standard deviation (*n* = 10) (**p < 0.01, ***p < 0.001, compared to the negative control group).

Micro-CT analysis revealed that the teeth of rats in the negative control group exhibited pit and fissure caries, with a low-density demineralized area beneath the mineralized layer in the teeth of rats in the NaF group. In contrast, no obvious demineralized area was observed in the sagittal plane of the SFN treatment group (Figure 10A). In comparison to the negative control group, each treatment group exhibited a significant increase in the bone volume fraction (BV/TV) (Figure 10B). However, only the 512 μg/mL SFN-treated group demonstrated a significant improvement in tooth mineral density (Figure 10C). Overall, our experiments demonstrated that SFN effectively attenuated the cariogenic virulence of *S. mutans* in the rat model of caries, controlling the occurrence and progression of caries *in vivo*.

### 3.8 *In vivo* biological safety

**Figure 11:**
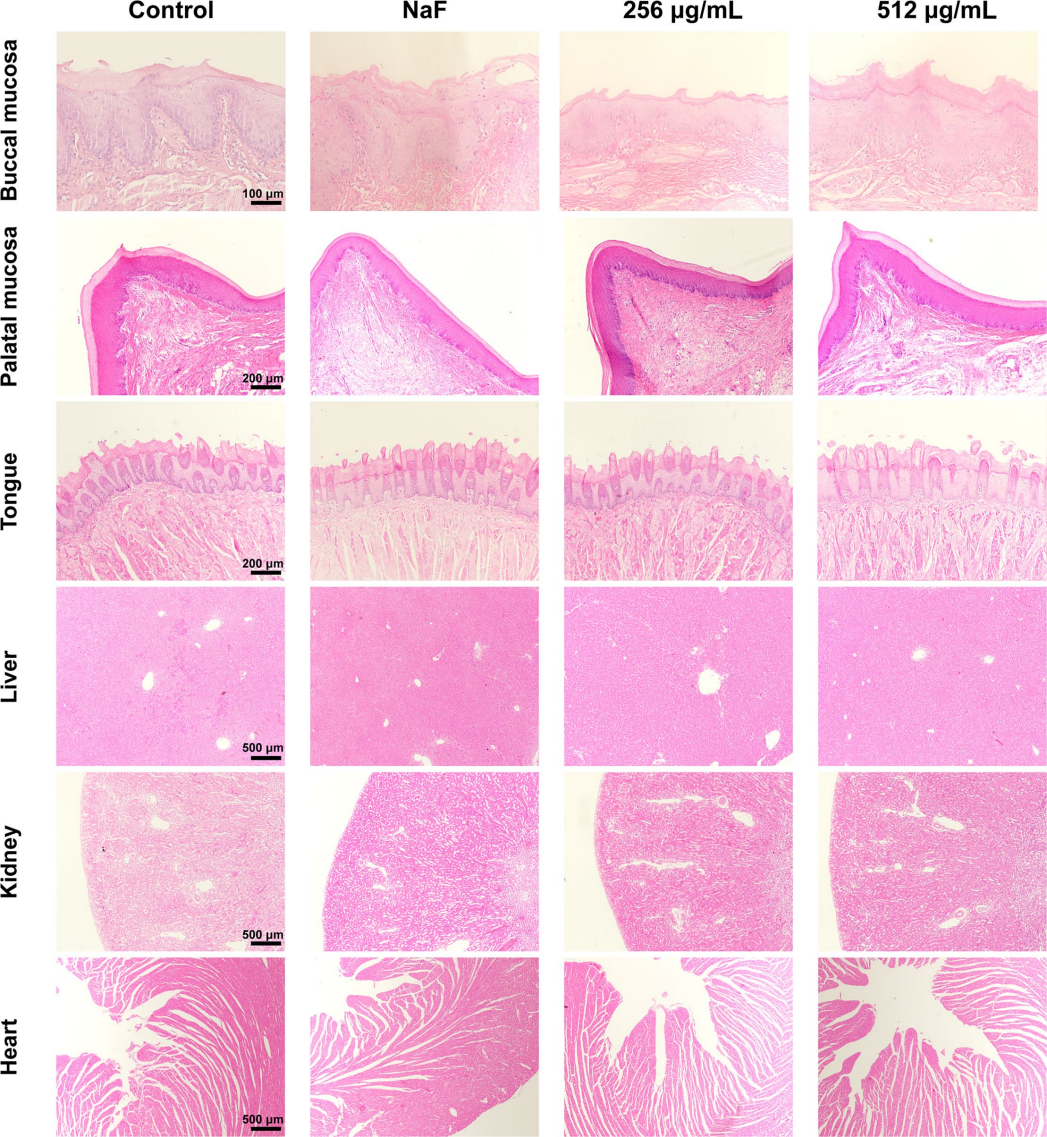
H&E staining of oral mucosa and organ tissues. No significant pathologic changes were found. Control means treated with 0.5% DMSO. NaF indicates a fluoride ion concentration of 1,000 ppm.

After the completion of the four-week treatment regimen, the rats were in good health, with no notable signs of congestion, edema, vesiculation, or ulcer formation in any of the oral mucosa. The in vivo safety of SFN was evaluated through staining of local mucosal and organ sections, as well as monitoring of body weight throughout the experimental period. No significant difference was detected in the weights of rats between the control and experimental groups (Figure 10D). Additionally, histological examination using hematoxylin–eosin (H&E) staining showed that the epithelium of the oral mucosa and organs in the SFN-treated group remained intact, with well-arranged cells. There were no pathological changes such as proliferative alterations, inflammation, reactive responses, or necrosis observed in comparison to the distilled deionized water (DDW) group (Figure 11). These findings indicated that SFN has excellent biocompatibility in vivo.

## 4 Discussion

As reported by Lancet in 2016, caries has the highest prevalence globally among all diseases, ranking second in morbidity after upper respiratory tract infections (Cooper, 2017). Given the emergence of drug-resistant bacterial strains and the increasing demand for drug biosafety, research on NPs and their potential as caries prevention agents has become essential in recent years (Porras et al., 2021; Tent et al., 2019). Numerous studies have demonstrated that SFN exhibits antimicrobial properties against various pathogenic bacteria, along with superior biosafety (Plaszkó et al., 2022; Krause et al., 2021; Li et al., 2022; Ghazizadeh-Hashemi et al., 2021). For the first time, we explored its effectiveness in controlling dental caries by inhibiting the primary cariogenic bacteria *S. mutans* with exciting findings, which is more conducive to understanding their structural and physicochemical characteristics and clarifying its biosafety.

Extensive evidence suggests that bacterial biofilms exhibit greater resistance to bacteriostatic agents compared to planktonic cells (Yan and Bassler, 2019). As a result, drug susceptibility tests may not provide an accurate reflection of the biological effects of SFN on *S. mutans*. It becomes crucial to explore the impact of SFN on *S. mutans* biofilms and cariogenic virulence. The growth curve results implied that the reduction in biofilm formation caused by SFN may be associated with its inhibitory effect on bacterial growth. Furthermore, our findings demonstrated that SFN dose-dependently inhibited biofilm formation and EPS synthesis of *S. mutans*. Notably, the inhibitory effect of SFN at 1/2MIC (128 μg/mL) is particularly significant. Although SFN did not exhibit a direct bactericidal effect on *S. mutans* at the experimental concentrations, however, there was statistically significant difference in CFU counts of viable bacteria after SFN treatment of pre-formed mature biofilms. Meanwhile, a substantial reduction in the extracellular matrix was still observed through SEM. The analysis on biofilm and structural imaging revealed that SFN disrupted the morphology and structure of *S. mutans* biofilm and bacterial surface. We hypothesized that SFN may have the potential to penetrate deeply into *S. mutans* biofilm and exert regulatory effects on the internal bacteria. Additionally, CLSM images showed that biofilms and EPS in groups treated with SFN appeared thin and scattered. Considering that EPS synthesis is crucial for the rapid adhesion of *S. mutans* to tooth surfaces (Zheng et al., 2023), we proposed that the inhibitory effect of SFN on the biofilm formation of *S. mutans* was closely linked to the reduction of water-insoluble EPS synthesis.

The ability to utilize carbohydrate metabolism to produce acid, ultimately leading to tooth demineralization, is a significant biological characteristic of *S. mutans* in the development of caries (Machiulskiene et al., 2020). Moreover, the capacity of *S. mutans* to survive in an acidic environment is a crucial prerequisite for the maintenance of the acidic environment on the surface of the teeth, and the sustained process of demineralization (Cheng et al., 2022). Sub-MIC levels of SFN exhibited a potent inhibitory effect on lactic acid production. Treatment with 64 μg/mL SFN significantly reduced the amount of acid production, while treatment with 128 μg/mL SFN almost completely inhibited acid production. However, a significant reduction was observed in all pH drop curves, especially during the initial phase, it is suggested that ambient temperature, distribution of solution media, and other relevant factors may exert an influence on the observed decrease in pH value. Nevertheless, 128 μg/mL SFN significantly inhibited the pH drop during *S. mutans* glycolysis and decreased the acid resistance of *S. mutans* in a lethal acidic environment.

The essential roles of genes *gtfB* and *gtfC* in the synthesis of EPS, cell aggregation, and adhesion have been well documented, promoting the formation of biofilms (Zheng et al., 2023). Additionally, the gene *gtfD* encodes for the production of water-soluble polysaccharides, which are involved in cellular metabolism (Zhang et al., 2021). In this study, we observed a significant downregulation in the expression of *gtfB*, *gtfC*, and *gtfD*, indicating an inhibition in both intracellular and extracellular polysaccharide synthesis. This inhibition was further supported by our observations of reduced biofilm formation and looser structures under SEM and CLSM. Additionally, the *atpD* gene, encoding the f_1_f_0_-ATPase, was found to be involved in maintaining the pH stability in both the internal and external environments of *S. mutans* (Smith et al., 1996). The qRT-PCR results demonstrated a significant downregulation in the expression of *atpD*, suggesting a notable decrease in acid tolerance. Furthermore, at higher sub-inhibitory concentrations, acid production was nearly undetectable, and a significant decrease in the expression of *ldh* was observed. This finding can be attributed to the impaired acid tolerance and disruption of biofilm structure, as *S. mutans* actively mitigates the adverse effects of low pH resulting from acid production. *S. mutans* engages in inter-bacterial communication and responds to diverse physiological and environmental stresses in a concerted manner through QS process (Suntharalingam and Cvitkovitch, 2005), thereby facilitating its biological behavior, including biofilm formation and the regulation of virulence factors, to adapt to changing environments (Kaur et al., 2015). The genes *VicR*, *comC*, *comD*, and *comE* are essential in promoting self-protection mechanisms in *S. mutans* when exposed to external stressors. Overexpression of *vicR* was shown to promote the expression of *gtfB*, *gtfC*, and *gtfD* (Senadheera et al., 2005). qRT-PCR analysis revealed varying degrees of repression in the transcriptional levels of these four genes. Interestingly, at the concentration of 32 μg/mL, we noticed an upregulation in the expression of *comC* and *comD*. This may be ascribed to the less potent inhibitory effect of low concentrations of SFN, which instead triggered the activation of genes closely linked to QS. As the concentration of SFN increased, a series of protective genes were suppressed and the expression of virulence genes were weakened. Consequently, SFN demonstrated significant efficacy in attenuating the cariogenic virulence of *S. mutans* without exerting sterilizing effects.

Due to the complex environment in the oral cavity, the inhibitory effect of SFN on *S. mutans* biofilm *in vivo* and its actual anticaries activities may not always consistent with the results *in vitro*. Moreover, in vitro cytocompatibility experiments are typically with short durations, making it challenging to identify potential toxic effects that may arise from prolonged slow exposure (Moghadam et al., 2020). Consequently, in vivo studies are crucial for conducting a thorough and meticulous assessment of the anti-caries effects and biosafety of SFN. In the present study, a rat caries model was established and employed to evaluate the roles of SFN in vivo. Among many studies, NaF has been used as a positive control due to its well-documented remineralization effect (Cai et al., 2021). In our study, the group treated with 1,000 ppm NaF exhibited more significant remineralization in the micro-CT images of sagittal plane and quantitative analysis of BV/TV. However, sub-surface demineralization of enamel still persisted, and caries lesions were observed in the fossa staining. This indicates that NaF alone was not effective in inhibiting caries development, despite its strong remineralization effects. In contrast, SFN at the MIC and 2MIC levels showed remarkable inhibition of caries occurrence and progression, as evidenced by significantly lower Keyes scores and fewer demineralization areas detected in the micro-CT analysis. Throughout the 4-week experiment, the rats in each treatment group remained in good general health, showing no significant side effects or toxic reactions. Tissue staining of oral mucosa and organ tissues revealed no morphologically or structurally abnormal cells. We further included SFN at a concentration of 512 μg/mL for additional observation of its caries prevention and biosafety. Rats treated with this concentration exhibited higher caries prevention activity than the negative control group, while maintaining normal physiological parameters. Importantly, this concentration was much lower than the safe concentration reported by Pogorzelska, A. et al. (Pogorzelska et al., 2023) and the concentration used in clinical trials (Ghazizadeh-Hashemi et al., 2021).

Previous studies have demonstrated that SFN exhibited inhibitory effects on a variety of pathogenic bacteria, including *S. aureus*, *B. anthracis*, enterohemorrhagic *E. coli*, and vancomycin-resistant enterococci, through diverse mechanisms. These mechanisms involve the suppression or modulation of bacterial biofilm formation, quorum sensing, enzyme activity, energy metabolism, and stress response (Nowicki et al., 2019; Nowicki et al., 2020; Deramaudt et al., 2020; Mazarakis et al., 2021; Cierpiał et al., 2020). Nonetheless, the exact mechanism through which SFN impedes *S. mutans* and the corresponding regulatory pathways remains unclear. The antibacterial effect of SFN on *S. mutans* appears to be complex, potentially involving the modulation of multiple enzyme activities, gene expression, and metabolic pathways. Further investigation is necessary to elucidate the precise mechanism and regulatory pathways underlying its inhibition of *S. mutans.*

To summarize, although SFN did not show the same robust direct bactericidal effect as CHX, it demonstrated excellent inhibiting effects on the cariogenic virulence of *S. mutans*, alongside a controlling effect against caries. SFN inhibited the metabolic activity of *S. mutans* biofilm and decreased the number of viable bacteria within the biofilm, without directly killing the cells. Owing to its ability to modulate and suppress the cariogenicity of *S. mutans*, along with prominent biosafety, SFN exhibited significant promise and developmental potential for future research in caries control.

## 5 Conclusion

Overall, the study demonstrated the outstanding antibacterial effect of SFN against *S. mutans* through inhibiting the growth of planktonic bacteria, disrupting the biofilm formation, and destroying biofilm structures. Sub-MIC levels of SFN effectively suppressed the ability of lactic acid production, acid resistance and EPS synthesis in a dose-dependent manner. Moreover, the expression of genes related to cariogenic virulence factors and quorum sensing were downregulated at sub-inhibitory concentration. The caries model in rats further displayed the prominent anti-caries effect of SFN with satisfactory biosafety. In this study, we conducted an initial investigation into the potential use of SFN as a natural anti-caries agent with excellent clinical and laboratory biosafety, which reveals a promising prospect in developing the novel approaches towards caries control. However, further studies in the regulatory mechanisms of SFN’s action on *S. mutans* and dental plaque multispecies biofilm, as well as its clinical application, are still needed.

## Data Availability

The datasets presented in this study can be found in online repositories. The names of the repository/repositories and accession number(s) can be found in the article/Supplementary material.
