# Supplementary material for: Sulforaphane as a promising anti-caries agents: inhibitory effects on Streptococcus mutans and caries control in a rat model
Source: Front Microbiol. 2025 Jan 3;15:1427803. doi: 10.3389/fmicb.2024.1427803 (PMC11738914; doi:10.3389/fmicb.2024.1427803)
Supplement: Supplementary file 1 [file Presentation_1.PDF]

Supplementary material

Supplementary Data (Original images of SEM)

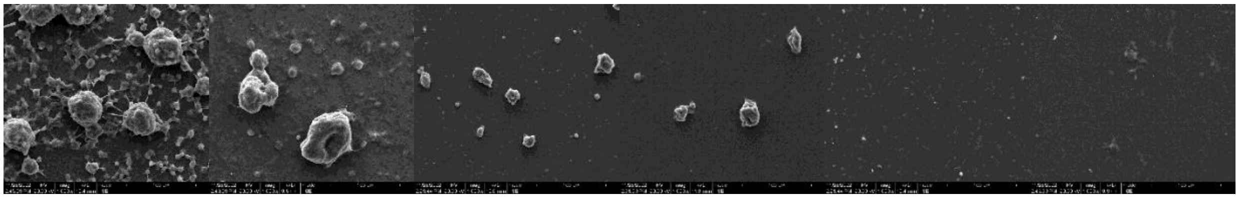

Figure S1 SEM images of *S. mutans* biofilms formation on coverslips captured at a magnification of 1,000×. From left to right: control， 32μg/mL, 64μg/mL, 128μg/mL, 256μg/mL, CHX.

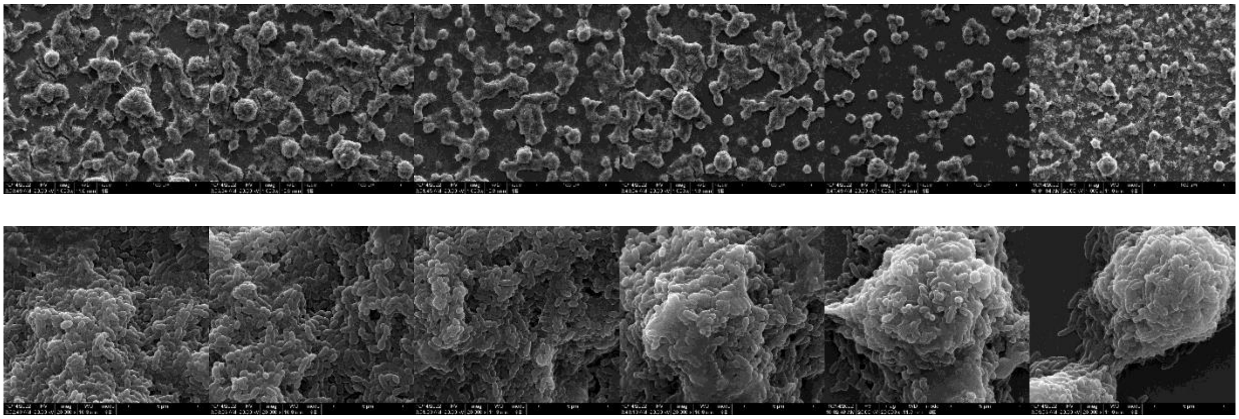

Figure S2 SEM images acquired at two different magnifications: 1,000× (upper) and 20,000× (nether) after the *S. mutans* biofilms exposed to various chemicals for 24 h. From left to right: control， 32μg/mL, 64μg/mL, 128μg/mL, 256μg/mL, CHX.
